# Cellular senescence: six decades of discovery and reinvention

**DOI:** 10.1038/s44318-026-00922-w

**Published:** 2026-10-02

**Authors:** Myrthe Klaver, Lotte Sophie Steeneken, Naomi Veeningen, Abdullah Altulea, Marco Demaria

**Affiliations:** European Research Institute for the Biology of Ageing (ERIBA), University of Groningen (RUG), University Medical Center Groningen (UMCG), Groningen, Netherlands

**Keywords:** Cancer, Cell Cycle, Molecular Biology of Disease

## Abstract

Cellular senescence has undergone a remarkable conceptual evolution since its discovery in 1961. Initially described by Hayflick and Moorhead as the finite proliferative lifespan of cultured human cells, senescence was first viewed as a consequence of cellular aging. The identification of telomere shortening, senescence biomarkers, and oncogene- and damage-induced senescence subsequently established its molecular basis and role as a tumor-suppressive stress response. The discovery of the senescence-associated secretory phenotype (SASP) further transformed the field by revealing that senescent cells actively communicate with and remodel their tissue environment. Genetic mouse models later demonstrated causal roles for senescent cells in tissue repair, aging, and age-related disease, while senotherapeutics established senescence as a therapeutic target. Here, we trace the major conceptual transitions that shaped the field, from replicative endpoint to stress-response program, regulator of tissue homeostasis, driver of chronic pathology, and clinically actionable process. We discuss the discoveries, controversies, and technological advances underlying these transitions and how emerging single-cell technologies, precision biomarkers, and targeted interventions are shaping the next era of senescence research.

## Abstract (web summary)

This review reflects on the field of cellular senescence and its continuing evolution in the context of aging and stress-response research, from initial discoveries to technology-driven conceptual transitions and the emerging era of targeted interventions.

## Graphical abstract

**Figure:**
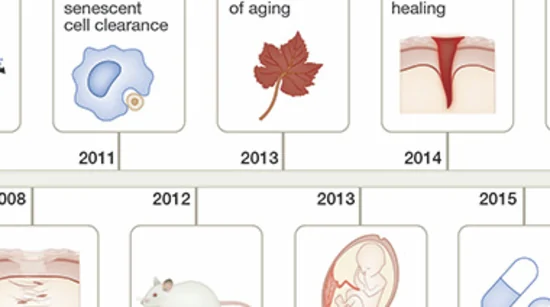

## The discovery of cellular senescence

In the early 20th century, it was widely assumed that physiological animal cells cultured in vitro possessed an unlimited proliferative capacity, provided that nutrients and culture conditions were continuously maintained. This view originated largely from the influential work of Alexis Carrel and became known as the Carrel dogma (Carrel, 1912). For nearly five decades, this concept shaped thinking about cellular proliferation, aging, and tissue maintenance.

**Figure 1:**
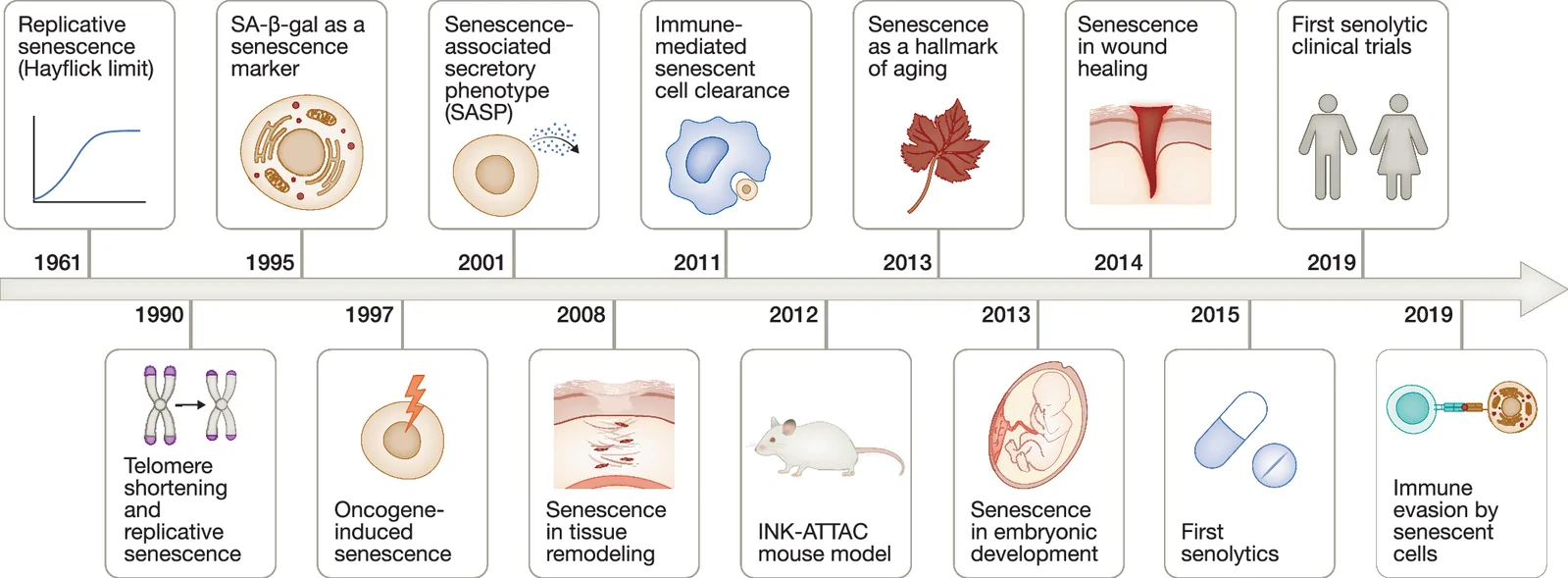
Major conceptual milestones in the evolution of cellular senescence. Timeline illustrating key discoveries that shaped the field, beginning with the Hayflick limit and replicative senescence, followed by the identification of telomere shortening, senescence biomarkers, oncogene-induced senescence, the senescence-associated secretory phenotype (SASP), genetic models enabling senescent-cell clearance, the development of senotherapeutics, and recent advances in senescence heterogeneity and precision targeting. Together, these milestones highlight the progressive transformation of senescence from a cell-culture phenomenon into a central age-associated mechanism and target for therapeutic intervention.

This paradigm was fundamentally challenged by the landmark studies of Hayflick and Moorhead, who demonstrated in 1961 that normal human fibroblasts undergo only a finite number of cell divisions before entering a state of irreversible growth arrest (Hayflick and Moorhead, 1961). This phenomenon, later termed the Hayflick limit, established the concept of replicative senescence and revealed that cellular proliferation is intrinsically constrained. More importantly, the discovery established replicative senescence as an experimental paradigm through which cellular mechanisms contributing to aging could be investigated. These foundational discoveries are summarized in Fig. 1, which highlights the major conceptual milestones that shaped the evolution of the field.

A major mechanistic breakthrough came nearly three decades later, when Harley and colleagues proposed that progressive telomere shortening underlies replicative senescence (Harley et al, 1990). Telomeres are repetitive DNA sequences that protect chromosome ends from degradation and inappropriate DNA repair. Because conventional DNA replication cannot fully replicate chromosome termini, telomeres progressively shorten with each cell division. Once a critical threshold is reached, dysfunctional telomeres trigger a persistent DNA damage response (DDR) that ultimately enforces stable cell-cycle arrest (d’Adda di Fagagna et al, 2003; Harley et al, 1990). The subsequent demonstration that ectopic expression of telomerase can bypass replicative senescence provided compelling experimental evidence that telomere erosion is a causal determinant of cellular lifespan. Together, these discoveries transformed senescence from a descriptive cellular phenomenon into a mechanistically tractable biological process.

## Defining and detecting senescent cells

### Emergence of biomarkers and molecular signatures

Once senescence became established as a biological phenomenon in cultured cells, a central question emerged: do senescent cells exist and accumulate in vivo? Addressing this challenge required the development of biomarkers capable of identifying senescent cells within tissues. The search for reliable senescence markers, therefore, became one of the major priorities of the field.

A major breakthrough came when Dimri et al, (1995) identified senescence-associated β-galactosidase (SA-β-Gal) as a practical histochemical marker of senescent cells in vitro and in aging tissues in vivo (Dimri et al, 1995). β-Galactosidase is present within lysosomes of both proliferating and senescent cells. However, senescent cells display increased lysosomal content and altered lysosomal physiology, resulting in elevated β-galactosidase activity detectable at the suboptimal pH of 6.0, a condition under which non-senescent cells show little activity. The SA-β-gal assay rapidly became the most widely used method for detecting senescent cells and, importantly, provided some of the first evidence that senescent cells accumulate in vivo. Nevertheless, it soon became apparent that increased lysosomal activity is not unique to senescence, highlighting the limitations of relying on a single marker.

The late 1990s also witnessed the identification of key molecular regulators of senescence. el-Deiry et al, (1993) identified p21 as a downstream effector of p53-mediated cell-cycle arrest in cancer cells (el-Deiry et al, 1993). Subsequently, Alcorta et al, (1996) demonstrated that p21 is strongly upregulated in senescent cells (Alcorta et al, 1996), while Serrano et al, (1997) established a central role for p16 in both senescence and tumor suppression (Serrano et al, 1997). Together, these studies defined the p53-p21 and p16–RB pathways as core molecular axes governing senescence and provided biomarkers that remain widely used today.

A further conceptual advance came with the recognition that persistent DNA damage signaling is a defining feature of many senescent cells. Studies investigating dysfunctional telomeres and oncogene-induced senescence revealed that senescence is frequently associated with a sustained DNA damage response (DDR) (Bartkova et al, 2006; d’Adda di Fagagna et al, 2003; Di Micco et al, 2006; Herbig et al, 2004). These findings linked replicative and stress-induced senescence through a common signaling framework and led to the identification of additional biomarkers, including γH2AX-positive DNA damage foci, 53BP1 foci, telomere dysfunction-induced foci (TIFs), and persistent activation of the p53-p21 pathway.

At the same time, senescent cells were found to undergo extensive chromatin remodeling. Narita et al, (2003) described the formation of senescence-associated heterochromatin foci (SAHF), specialized chromatin domains that contribute to the stable repression of proliferation-associated genes (Narita et al, 2003). These observations reinforced the emerging view that senescence is not a passive loss of proliferative capacity, but an actively maintained cellular state involving extensive molecular reprogramming.

Perhaps the most important lesson emerging from three decades of biomarker discovery is that senescence cannot be defined by any single feature. Instead, it is best understood as a composite phenotype characterized by stable cell-cycle arrest, stress signaling, chromatin remodeling, altered metabolism, secretory activity, and context-dependent molecular markers. Recent advances in single-cell transcriptomics, spatial profiling, and high-dimensional phenotyping have further highlighted the limitations of single-marker approaches and revealed substantial heterogeneity among senescent cells. Consequently, the field is increasingly moving away from defining senescence through individual biomarkers and toward identifying multidimensional senescence states.

## Inducers and mechanisms of senescence

**Figure 2:**
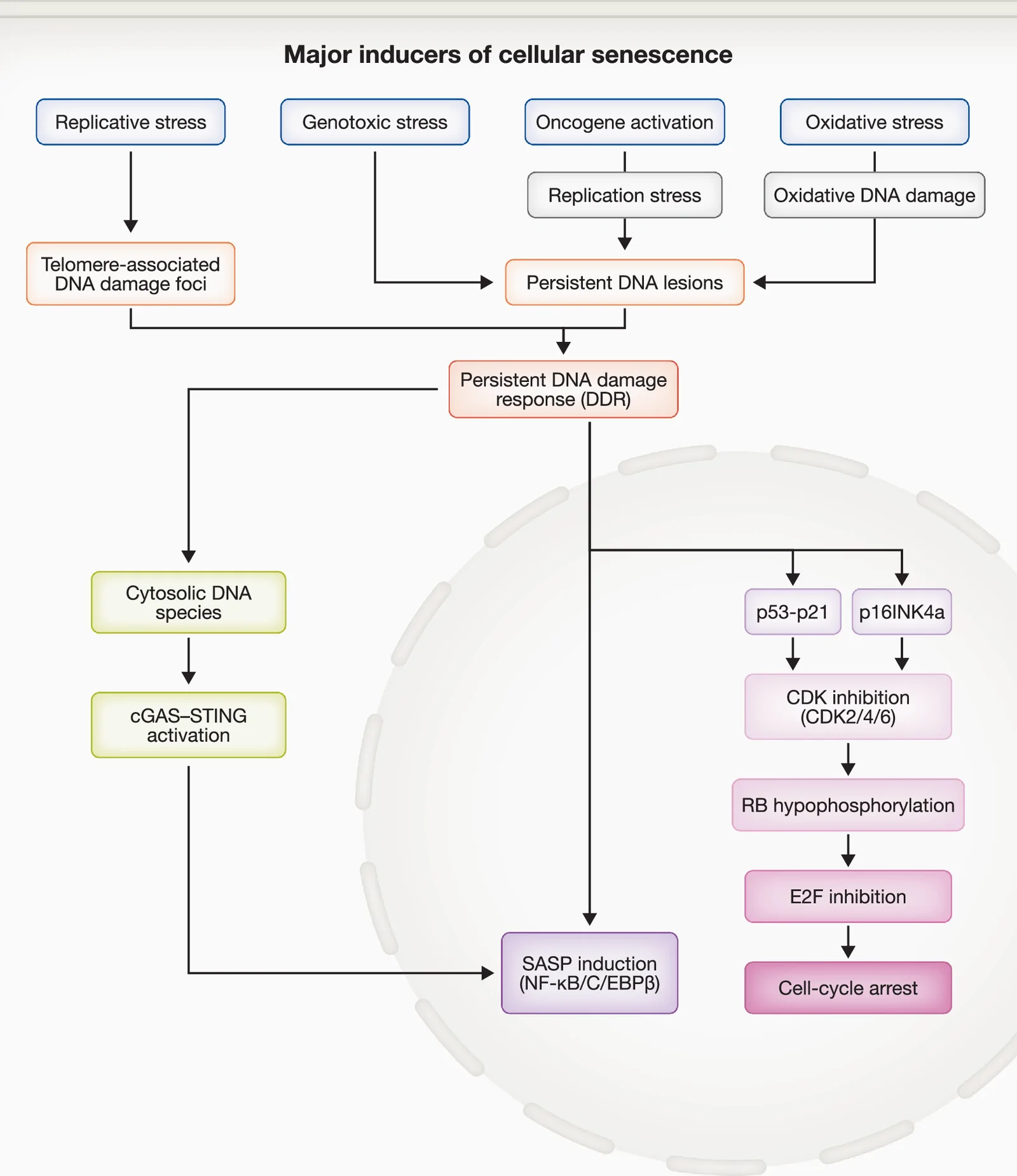
Major pathways leading to cellular senescence and SASP activation. Diverse stressors, including telomere attrition, oncogenic signaling, genotoxic damage, oxidative stress, and mitochondrial dysfunction, converge on core senescence-regulatory pathways centered on p53-p21 and p16–RB signaling. Persistent stress signaling activates multiple SASP regulatory networks, including NF-κB, p38 MAPK, mTOR, and cGAS-STING pathways, generating context-dependent secretory programs that influence tissue remodeling, immune surveillance, and disease progression.

The discovery of replicative senescence initially linked senescence to cellular aging. However, subsequent studies revealed that similar phenotypes could arise independently of replicative exhaustion in response to diverse forms of cellular stress. The realization that senescence could be induced without telomere shortening represented one of the most important conceptual advances in the history of the field. It suggested that senescence is not merely a consequence of aging, but a broader biological program activated by cellular damage and aberrant signaling (Fig. 2).

### Oncogene-induced senescence (OIS)

A major breakthrough came in 1997 when Serrano et al demonstrated that oncogenic RAS signaling induces senescence in primary human and rodent cells (Serrano et al, 1997). This phenomenon, termed oncogene-induced senescence (OIS), transformed senescence from a presumed consequence of cellular aging into an active stress-response program that functions as a barrier to malignant transformation. In this seminal study, RAS activation induced stable G1 cell-cycle arrest accompanied by characteristic morphological changes and the induction of senescence-associated features, including increased expression of p53, p21, p16, SA-β-gal activity, and plasminogen activator inhibitor type I (PAI-1) (Serrano et al, 1997).

Importantly, Serrano and colleagues also identified species-specific differences in the regulation of OIS. In rodent cells, disruption of either p53 or p16 was sufficient to bypass senescence, whereas human cells required simultaneous inactivation of both pathways, highlighting the complexity of senescence regulation in different biological contexts (Serrano et al, 1997).

Shortly after the discovery of RAS-induced senescence, two follow-up studies demonstrated that activation of the Raf-MEK-ERK pathway was likewise sufficient to induce senescence, indicating that OIS reflects a broader response to aberrant mitogenic signaling rather than the activity of a single oncogene (Lin et al, 1998; Zhu et al, 1998). Subsequent work expanded this concept to additional oncogenic pathways, including PI3K-AKT signaling (Astle et al, 2012; Kuilman et al, 2008; Wajapeyee et al, 2008; Xu et al, 2014). In parallel, investigations into the underlying mechanisms revealed a central role for DNA damage signaling. Activation of oncogenes was shown to induce replication stress and persistent DNA damage responses, while inhibition of key DDR components such as ATM impaired senescence induction following oncogenic H-RASV12 activation (Bartkova et al, 2006; Di Micco et al, 2006; Mallette et al, 2007). Together, these studies established OIS as a general response to aberrant proliferative signaling rather than a pathway-specific phenomenon. Although OIS was initially viewed as a relatively uniform tumor-suppressive program, recent work has revealed greater complexity. The concept of an OIS spectrum proposes that OIS comprises heterogeneous cellular states rather than a single uniform endpoint (Chan et al, 2024).

Collectively, these discoveries fundamentally redefined senescence biology by positioning senescence as an intrinsic tumor-suppressive mechanism and one of the first examples of a cellular response capable of actively preventing malignant transformation. More recent recognition of OIS heterogeneity has further refined this paradigm, emphasizing that the biological consequences of OIS are highly context dependent.

### Genotoxic and stress-induced senescence

The discovery of OIS was followed by another major conceptual shift: the recognition that senescence can be triggered by a broad range of damaging stimuli. Senescence was no longer viewed as a response exclusive to telomere attrition or oncogenic signaling, but increasingly recognized as a general response to cellular stress. A wide variety of genotoxic and environmental insults can induce senescence, often converging on common signaling pathways despite distinct initiating events (Fig. 2).

The senescence-inducing effects of genotoxic stress were first described in the early 1990s. Di Leonardo et al demonstrated that γ-irradiation induces permanent cell-cycle arrest accompanied by the characteristic enlarged and flattened morphology associated with senescent cells (Di Leonardo et al, 1994). Subsequent studies showed that other forms of DNA damage, including X-rays, ultraviolet radiation, and chemotherapeutic agents such as bleomycin, actinomycin D, and doxorubicin, similarly induce senescence-associated phenotypes (Chang et al, 1999; Hong et al, 2010; Latonen et al, 2001; Robles and Adami, 1998; Suzuki et al, 2001; Ungvari et al, 2013). These observations established therapy-induced senescence and DNA damage-induced senescence as important biological responses to genotoxic stress.

At the molecular level, many of these stimuli converge on persistent activation of the DNA damage response (DDR), which promotes stable cell-cycle arrest through the p53-p21 and RB pathways (d’Adda di Fagagna, 2008). The recognition that replicative senescence, OIS, and genotoxic stress-induced senescence share common DDR-associated signaling mechanisms provided an important conceptual framework linking previously distinct senescence-inducing stimuli.

In parallel, studies of oxidative stress revealed that senescence can also arise in the absence of direct DNA lesions. Modulation of oxidative stress through H₂O₂ treatment or hyperoxic culture conditions was shown to induce proliferative arrest and shorten cellular lifespan (Chen and Ames, 1994; Chen et al, 1995). These findings led to the concept of stress-induced premature senescence (SIPS), which describes senescence triggered by sublethal environmental stressors rather than extensive genomic damage (Toussaint et al, 2000). Subsequent work demonstrated that reactive oxygen species (ROS), particularly those generated by dysfunctional mitochondria, contribute to senescence through both DNA damage-dependent and DNA damage-independent mechanisms (Hou et al, 2022; Parrinello et al, 2003; Passos et al, 2007; Wiley et al, 2016).

Together, these discoveries marked a major conceptual transition in the field. Senescence was no longer viewed solely as the endpoint of replicative aging but increasingly recognized as a versatile stress-response program activated by diverse forms of cellular damage and dysfunction. This expanded framework laid the foundation for understanding senescence as a central mechanism linking cancer, tissue repair, aging, and age-related disease.

## Senescence-associated secretory phenotype

Cellular senescence is often viewed as a classic example of antagonistic pleiotropy, whereby a process that promotes tissue homeostasis and tumor suppression early in life contributes to aging and disease later in life (Kirkwood and Austad, 2000). While senescent cells were initially recognized for their ability to enforce stable growth arrest and suppress malignant transformation, accumulating evidence also linked them to chronic inflammation, tissue dysfunction, and age-related pathology (Baker et al, 2016; Baker et al, 2011; Kang et al, 2011; Laberge et al, 2015). For many years, the basis for these seemingly opposing effects remained unclear.

A major conceptual breakthrough came with the realization that senescent cells are not biologically inert. Instead, they actively communicate with their surroundings through the secretion of soluble and vesicular factors capable of influencing neighboring cells and reshaping tissue microenvironments. This discovery fundamentally transformed the field, redefining senescence from a purely cell-autonomous growth arrest program into a non-cell-autonomous regulator of tissue biology.

The first evidence for this concept came from the laboratory of Judy Campisi, who demonstrated that senescent fibroblasts stimulate the proliferation of preneoplastic epithelial cells while having little effect on normal epithelial cells (Krtolica et al, 2001). These observations suggested that senescent cells exert biological effects, extending far beyond their own proliferative arrest. Through a series of influential studies, the senescence-associated secretory phenotype (SASP) emerged as a defining feature of senescence and a major determinant of its physiological and pathological consequences (Acosta et al, 2008; Basisty et al, 2020; Coppé et al, 2008; Kuilman et al, 2008; Kuilman and Peeper, 2009). Importantly, SASP expression was shown to be dynamic rather than constitutive, with most factors appearing several days after senescence induction and displaying kinetics that vary according to the inducing stimulus (Coppé et al, 2008).

Initially, the SASP was largely defined as a collection of secreted cytokines, chemokines, growth factors, and matrix-remodeling enzymes identified using antibody-based approaches and biochemical analyses of conditioned media (Coppé et al, 2008; Glück et al, 2017; Mosteiro et al, 2016). However, advances in transcriptomics, proteomics, and metabolomics progressively expanded this view. Many SASP components were found to be regulated at the transcriptional level (Hernandez-Segura et al, 2017; Kohli et al, 2021), while unbiased profiling approaches revealed a substantially greater complexity than initially appreciated (Basisty et al, 2020; Özcan et al, 2016). The SASP is now understood as a multidimensional secretome that extends beyond proteins to include bioactive lipids, such as leukotrienes and prostaglandins (Cheng et al, 2021; Dackor et al, 2011; Wiley et al, 2019), as well as extracellular vesicles carrying proteins, lipids, microRNAs, and other non-coding nucleic acids (Borghesan et al, 2019; Takasugi et al, 2017).

A further conceptual shift emerged with the recognition that there is no single universal SASP. Instead, senescent cells generate highly diverse secretory programs that vary according to cell type, tissue context, senescence inducer, and underlying signaling pathways (Basisty et al, 2020; Özcan et al, 2016). This heterogeneity helps explain the remarkably diverse biological effects attributed to senescent cells. For example, certain p53-p21-associated secretory programs promote immune surveillance and facilitate the clearance of senescent or damaged cells (Sturmlechner et al, 2021; Wang et al, 2022; Xue et al, 2007), whereas other SASP programs are dominated by pro-inflammatory and pro-tumorigenic factors that contribute to chronic disease (Chien et al, 2011; Crescenzi et al, 2011; Kolesnichenko et al, 2021).

Mechanistically, NF-κB and C/EBPβ emerged as the first major transcriptional regulators of inflammatory SASP programs (Acosta et al, 2008; Chien et al, 2011; Kuilman et al, 2008). Subsequent studies demonstrated that persistent DNA damage signaling, through molecules such as ATM, NBS1, CHK2, ATR, GATA4, and PARP1, contributes to SASP establishment and maintenance (Kang et al, 2015; Rodier et al, 2009). Additional pathways, including p38 MAPK and mTOR signaling, were later shown to regulate SASP expression either directly or through modulation of NF-κB activity (Freund et al, 2011; Herranz et al, 2015; Laberge et al, 2015).

However, the growing diversity of SASP profiles revealed that SASP regulation cannot be explained solely through NF-κB signaling. The cGAS-STING pathway emerged as an additional mechanism linking persistent DNA damage to inflammatory and interferon responses through cytoplasmic DNA sensing, while more recent work identified mitochondrial double-stranded RNA sensing via the RIG-I/MDA5-MAVS pathways as a complementary regulator of SASP expression (Dou et al, 2017; Glück et al, 2017; López-Polo et al, 2024; Yang et al, 2017). At the same time, studies identified alternative SASP programs driven by p53, p16, and NOTCH signaling. For example, p53 accumulation or prolonged CDK4/6 inhibition can generate secretory profiles enriched in p53-regulated proteins and type-I interferon signaling (Coppé et al, 2011; Wang et al, 2022; Wiley et al, 2018), whereas NOTCH activation promotes a TGF-β-dominated secretome with relatively limited inflammatory activity (Hoare et al, 2016).

Together, these discoveries transformed the understanding of senescence. Rather than representing a simple state of proliferative arrest, senescence is now viewed as a dynamic and context-dependent tissue-remodeling program whose biological effects are largely mediated through diverse secretory phenotypes. The recognition of SASP heterogeneity has also provided a conceptual tool for understanding how senescent cells can exert both beneficial and detrimental effects in different physiological and pathological contexts.

## In vivo evidence and physiological roles

### Demonstrating functional roles of senescent cells in vivo

For several decades, evidence linking senescent cells to aging and disease remained largely correlative. Although senescent cells were increasingly observed in aged tissues and pathological conditions, it remained unclear whether they actively contributed to these processes or simply accumulated as a consequence of tissue dysfunction.

A major breakthrough came with the development of genetic models that enabled selective identification and elimination of senescent cells in vivo. The first widely used example was the INK-ATTAC mouse, in which p16-positive cells can be selectively ablated through inducible apoptosis (Baker et al, 2011). Using this system, Baker and colleagues demonstrated that senescent-cell clearance delays age-associated pathology in progeroid mice (Baker et al, 2011), while subsequent studies showed improvements in healthspan and lifespan in naturally aged animals (Baker et al, 2016). These experiments provided some of the first direct evidence that senescent cells can contribute causally to organismal aging.

Additional models, including p16-3MR (Demaria et al, 2014), Glb1-based reporters (Sun et al, 2022), and p21-based systems (Chandra et al, 2022; Yi et al, 2023), expanded the ability to track and manipulate senescent cells in vivo. As with other reporter systems, differences in model design and experimental conditions can lead to discrepancies between studies, as highlighted by a recent debate concerning the p16-3MR model (Hori et al, 2026; Wang et al, 2026). More recently, lineage-specific approaches have revealed substantial heterogeneity among senescent populations and demonstrated that senescent cells arising from different tissues and cellular lineages can exert distinct biological functions (Farr et al, 2023; Nishikawa et al, 2026; Suda et al, 2025; Zhao et al, 2024).

Collectively, these studies transformed senescence research from descriptive observation into experimental biology. Rather than simply documenting the presence of senescent cells, investigators could now directly test their physiological functions and determine when senescence contributes to tissue homeostasis, aging, or disease.

### Senescence in tissue repair

While genetic models established that senescent cells can contribute to aging and disease, a parallel series of discoveries challenged the emerging perception that senescence is inherently detrimental. Instead, senescence was increasingly recognized as a physiological process involved in tissue remodeling and repair.

The first evidence for such a role came from studies of liver fibrosis. In a landmark study, Krizhanovsky et al demonstrated that activated hepatic stellate cells undergo senescence during fibrosis and that this response limits excessive extracellular matrix deposition (Krizhanovsky et al, 2008). Rather than promoting tissue dysfunction, senescence acted as a protective mechanism that restricted fibrotic progression. These findings introduced the idea that senescence can contribute positively to tissue homeostasis by terminating potentially harmful cellular programs.

Shortly thereafter, similar observations were reported in cutaneous wound healing. Jun et al demonstrated that senescent fibroblasts accumulate transiently during tissue repair and contribute to the resolution of fibrosis (Jun and Lau, 2010). These studies suggested that senescence is not simply a response to damage, but can actively participate in orchestrating regenerative processes.

Direct evidence for a functional role of senescent cells in tissue repair came from studies using genetic ablation models. Demaria et al demonstrated that selective elimination of senescent cells delays wound closure and impairs tissue regeneration (Demaria et al, 2014). Mechanistically, senescent fibroblasts were shown to promote myofibroblast differentiation through the secretion of PDGF-AA, thereby accelerating wound healing. Importantly, this study established that senescent cells are not merely present during tissue repair but actively contribute to successful regeneration. Subsequent studies extended these findings across vertebrate and invertebrate organisms, demonstrating functional roles for senescence in tissue regeneration and responses to injury, including during limb and fin regeneration (Da Silva-Álvarez et al, 2020a; Perrigue et al, 2015; Salinas-Saavedra et al, 2023; Yun et al, 2015). Nevertheless, in certain models, such as planarians, the evidence remains limited, and additional markers as well as further research are required before the role of senescence can be conclusively established. These observations also provided a new perspective on the biological significance of the SASP. Rather than functioning solely as a source of chronic inflammation, transient SASP production emerged as a mechanism through which senescent cells coordinate tissue remodeling by influencing neighboring stromal, epithelial, immune, and progenitor cells. In this context, senescence can be viewed as a temporary signaling state that facilitates communication between damaged tissues and the cellular programs required for their repair.

Together, these studies fundamentally altered the perception of senescence. Instead of representing exclusively a tumor-suppressive or pathological endpoint, senescence emerged as a physiological component of tissue remodeling. This realization helped explain why a process capable of promoting aging and chronic disease has been evolutionarily conserved.

### Senescence in development

An even more unexpected conceptual advance emerged in 2013, when two independent groups reported the presence of senescent cells during embryonic development (Muñoz-Espín et al, 2013; Storer et al, 2013). These findings, together with complementary evidence from Chuprin et al demonstrating the role of senescence in embryonic tissue remodeling (Chuprin et al, 2013), challenged the prevailing view that senescence was solely a response to cellular stress, DNA damage, oncogenic signaling, or aging-associated dysfunction. Instead, they suggested that senescence may represent a fundamental biological program that extends beyond damage responses.

Both studies identified cells exhibiting senescence-associated features within transient embryonic structures, including the mesonephric tubules, endolymphatic sac, and apical ectodermal ridge (Muñoz-Espín et al, 2013; Storer et al, 2013). These cells displayed characteristic hallmarks of senescence, including proliferative arrest, SA-β-gal activity, and expression of p21. Importantly, they were subsequently eliminated by infiltrating macrophages, suggesting that programmed senescence and immune-mediated clearance cooperate during tissue remodeling. Subsequent studies identified developmental senescence in additional vertebrate species, including birds, amphibians and fish, suggesting that senescence during embryogenesis represents an evolutionarily conserved program rather than a species-specific phenomenon (Da Silva-Álvarez et al, 2020b; Davaapil et al, 2017; Nacher et al, 2006; Villiard et al, 2017).

Developmental senescence differs in several important ways from classical stress-induced senescence. Whereas damage-induced senescence is frequently associated with persistent DNA damage signaling and activation of the p16 and p53 pathways, developmental senescence is largely dependent on p21 and occurs independently of both p16 and p53 (Muñoz-Espín et al, 2013; Storer et al, 2013). These findings demonstrated that senescence can be initiated through distinct molecular mechanisms while generating a similar phenotypic outcome.

The discovery of developmental senescence had important evolutionary implications. It suggested that senescence may not have evolved solely as a tumor-suppressive mechanism or as a consequence of aging, but may instead represent an ancient tissue-remodeling program that was subsequently adapted for additional biological functions. This realization provided an important basis for understanding the diverse physiological roles of senescence and further reinforced the view that senescence is fundamentally a context-dependent process. Together with studies of tissue repair and regeneration, these discoveries also helped resolve the apparent paradox of why a process capable of driving aging and disease has been evolutionarily conserved.

## Senescence in aging and disease

### Senescence and aging

The discovery of beneficial roles for senescence in development and tissue repair raised a fundamental question: how does a physiological tissue-remodeling program become a driver of aging and disease? A major clue came from observations that senescent cells accumulate with advancing age across multiple tissues and species.

Beginning in the 1990s and early 2000s, numerous studies reported increased abundance of senescence-associated markers in aged tissues, including SA-β-gal activity, p16 expression, persistent DNA damage signaling, and other senescence-associated features. Senescent cells were identified in multiple organs, including skin, adipose tissue, liver, kidney, lung, and the hematopoietic system, suggesting that senescence is a widespread consequence of aging. These observations led to the hypothesis that the gradual accumulation of senescent cells may contribute to the progressive decline in tissue function observed during aging.

Initially, however, the significance of these findings remained controversial. Senescent cells could simply represent a biomarker of accumulated damage rather than a causal driver of aging. The field, therefore, faced a challenge similar to that encountered in cancer biology decades earlier: distinguishing correlation from causation.

As evidence accumulated, senescence increasingly emerged as a unifying mechanism capable of linking many established drivers of aging. Telomere dysfunction, genomic instability, mitochondrial dysfunction, epigenetic alterations, stem-cell exhaustion, and chronic inflammation can all induce or reinforce senescence programs. This central position within aging biology contributed to the incorporation of cellular senescence into the Hallmarks of Aging framework (Lopez-Otin et al, 2013), where it was recognized as one of the fundamental biological processes contributing to organismal aging. Its inclusion further accelerated interest in understanding how senescent cells influence tissue function throughout life.

The strongest evidence for a causal role of senescence came from genetic ablation studies. Using INK-ATTAC mice, Baker and colleagues demonstrated that removal of p16-positive cells delays age-associated pathology in progeroid animals (Baker et al, 2011). Subsequent studies extended these findings to naturally aged mice and showed improvements in healthspan and lifespan following senescent-cell clearance (Baker et al, 2016). However, these effects were not universally reproduced across senescent-cell ablation models. An independent p16-lineage tracing and ablation study reported that elimination of p16-expressing cell lineages can have detrimental effects on liver function, highlighting the context dependence of senescent cell contributions to aging (Grosse et al, 2020). These experiments fundamentally changed the field by demonstrating that senescent cells are not merely passive indicators of aging but active contributors to age-related decline, while also revealing that their effects can depend on the cellular population targeted and the experimental context.

A conceptual shift emerged from these observations. Senescence appears beneficial when induced transiently and efficiently cleared, contributing to tissue remodeling and repair. However, when senescent cells persist because of continuous damage, repeated stress exposure, or inefficient immune-mediated clearance, they accumulate and exert detrimental effects on tissue homeostasis. Aging can therefore be viewed, at least in part, as a progressive imbalance between the generation and removal of senescent cells.

This new mechanism helped reconcile the apparent paradox of senescence: a biological process that evolved to protect tissues and maintain homeostasis can, when persistent, become a major driver of aging-associated dysfunction.

### SASP as a driver of chronic disease

As senescent cells became increasingly implicated in age-related disorders, attention shifted from determining whether senescence occurs in disease to understanding how senescent cells drive pathology. A central role emerged for the SASP, which provided a mechanistic explanation for how relatively small numbers of senescent cells can exert profound effects on tissue function.

The first evidence came from cancer biology. Krtolica et al demonstrated that senescent fibroblasts stimulate the proliferation of preneoplastic epithelial cells, revealing that senescent cells can influence neighboring cells through paracrine signaling (Krtolica et al, 2001). Subsequent studies identified numerous SASP factors, including IL-6, IL-8, CCL5, HGF, MMPs, VEGF, CXCL5, CXCL12, and soluble E-cadherin, that promote tumor growth, angiogenesis, invasion, and metastasis (Ancrile et al, 2007; Chen et al, 2019; Coppé et al, 2006; Kawaguchi et al, 2021; Kim et al, 2017; Liu and Hornsby, 2007; Luo et al, 2016; Ortiz-Montero et al, 2017). These findings established a paradox that remains central to the field: while senescence suppresses malignant transformation at the cellular level, persistent senescent cells can promote tumor progression at the tissue level.

Beyond cancer, chronic SASP signaling emerged as a common mechanism linking senescence to a broad spectrum of age-related diseases. In cardiovascular and metabolic disorders, SASP factors, including IL-1β, IL-6, TNF, MCP-1, and MMPs, promote immune-cell recruitment, vascular inflammation, atherosclerotic plaque progression, and insulin resistance (Childs et al, 2016; Cui et al, 2018; Dookun et al, 2020; Gardner et al, 2015; Honda et al, 2021; Rippe et al, 2012; Zhou et al, 2006). In musculoskeletal and fibrotic diseases, inflammatory cytokines, matrix-remodeling enzymes, leukotrienes, and pro-fibrotic mediators disrupt tissue regeneration and drive pathological extracellular matrix remodeling, contributing to osteoporosis as well as pulmonary, liver, and cardiac fibrosis (Álvarez et al, 2017; Bird et al, 2018; Ferreira-Gonzalez et al, 2018; Kapoor et al, 2011; Ma et al, 2015; Ramadhiani et al, 2021; Ritschka et al, 2020; Schafer et al, 2017; Wei et al, 2018; Wiley et al, 2019).

Similar concepts have emerged in neurodegenerative disease. Senescence-associated inflammatory signaling contributes to neuroinflammation, protein aggregation, and impaired neuronal function, while pharmacological suppression of key SASP regulators such as p38 MAPK and NF-κB alleviates pathological features in experimental models (Gaikwad et al, 2021). These findings further support the idea that chronic senescence can influence tissue function well beyond the cells in which it originates.

Collectively, these observations transformed the understanding of age-related disease. Rather than acting solely through cell-autonomous dysfunction, senescent cells emerged as active drivers of pathology capable of reshaping tissue microenvironments through chronic inflammatory and remodeling signals. The SASP, therefore, provided a unifying mechanism linking senescence to multiple age-related diseases and helped explain how a physiological tissue-remodeling program can become a source of chronic degeneration when senescent cells persist.

## Senescence and immune regulation

The discoveries that senescence contributes to tissue repair, development, aging, and chronic disease raised a fundamental question: what determines whether senescent cells are transiently beneficial or persist long enough to become detrimental? Increasing evidence suggests that the answer lies, at least in part, in their interactions with the immune system.

Although senescence was initially viewed as a largely cell-autonomous process governed by intracellular stress-response pathways, it is now recognized as a highly dynamic state regulated by continuous communication with immune cells. Senescent cells actively recruit, activate, and modulate components of both the innate and adaptive immune system through the SASP and other surface-associated signals. In turn, immune cells can recognize and eliminate senescent cells, thereby limiting their accumulation and restoring tissue homeostasis.

This reciprocal relationship has led to the concept of senescence surveillance, whereby immune-mediated clearance serves as a critical mechanism controlling senescent-cell burden. Conversely, failure of immune surveillance allows senescent cells to persist and accumulate, contributing to aging and age-related disease.

### Immune surveillance of senescent cells

The first direct evidence that senescent cells are actively monitored and eliminated by the immune system came from the landmark study by Xue et al, (2007), who demonstrated that restoration of p53 activity in liver tumors induces cellular senescence and triggers tumor regression through an immune-dependent mechanism (Xue et al, 2007). Senescent tumor cells recruited innate immune populations, including NK cells, macrophages, and neutrophils, resulting in their elimination from the tissue. This study fundamentally altered the perception of senescence by demonstrating that senescent cells are not necessarily permanent residents of tissues, but can be actively removed through coordinated immune responses.

Subsequent studies expanded the range of immune populations implicated in senescent-cell clearance. Kang et al demonstrated that CD4⁺ T cells contribute to the elimination of senescent hepatocytes through mechanisms requiring monocyte and macrophage recruitment (Kang et al, 2011). In parallel, Sagiv et al showed that NK cells directly eliminate senescent cells through perforin- and granzyme-dependent cytotoxicity (Sagiv et al, 2013). Together, these findings established that both innate and adaptive immune compartments participate in senescence surveillance.

A major advance came with the realization that the SASP is not only a mediator of tissue remodeling but also a key regulator of immune recruitment. Iannello et al demonstrated that p53-induced senescence promotes secretion of chemokines, including CCL2, CCL3, CCL4, and CCL5, with CCL2 playing a particularly important role in NK-cell recruitment (Iannello et al, 2013). Effective clearance further depended on interactions between the activating receptor NKG2D and its ligands expressed on senescent cells, as blockade of NKG2D delayed tumor elimination (Iannello et al, 2013).

Additional studies identified other factors linking senescence and immunity. Davalos et al demonstrated that senescent cells release HMGB1, a damage-associated molecular pattern that promotes recruitment and activation of macrophages and NK cells (Davalos et al, 2013). More broadly, accumulating evidence revealed that senescent cells utilize a diverse repertoire of cytokines, chemokines, growth factors, and alarmins to shape their surrounding immune microenvironment and facilitate their own recognition and clearance.

Together, these discoveries established immune surveillance as a central component of senescence biology. Rather than representing a static endpoint of cellular stress, senescence emerged as a dynamic process in which cellular fate is strongly influenced by interactions with the immune system.

### Immune evasion and senescent cells persistence

The recognition that senescent cells are subject to immune surveillance immediately raised another fundamental question: if senescent cells can be efficiently recognized and eliminated, why do they progressively accumulate during aging and persist in chronic diseases? Addressing this paradox led to a major conceptual advance in the field: senescent cells are not passive targets of immune clearance but can actively evade immune surveillance.

The first direct evidence for this phenomenon came from Pereira et al, (2019), who demonstrated that senescent cells upregulate the non-classical MHC molecule HLA-E following senescence induction (Pereira et al, 2019). HLA-E was found to be highly expressed in senescent cells and to interact with the inhibitory receptor NKG2A on NK cells and CD8⁺ T cells, thereby suppressing cytotoxic activity and impairing immune-mediated clearance (Ljubič et al, 2023; Wada et al, 2004). These findings established that senescent cells can exploit immune checkpoint-like pathways to protect themselves from elimination.

This discovery fundamentally changed the understanding of senescence persistence. Rather than reflecting only a decline in immune function, senescent-cell accumulation could also result from active adaptations that reduce immune recognition. In this view, senescent cells and immune cells engage in a dynamic interaction in which immune surveillance is continuously counterbalanced by immune-evasion mechanisms.

Additional pathways have since been proposed. Molecules such as Galectin-9 and CEACAM1 can interact with the inhibitory receptor TIM-3, a pathway known to suppress NK-cell and T-cell activity in chronic inflammation and cancer (Anderson et al, 2016; Huang et al, 2015). Likewise, HMGB1, initially characterized as a pro-inflammatory danger signal that promotes immune recruitment, can exert immunosuppressive effects depending on its redox state and local microenvironment (Kang et al, 2014; Venereau et al, 2012). These observations illustrate how the same molecules that initially facilitate immune recognition may, under different conditions, contribute to immune dysfunction and persistence.

Other candidate mechanisms continue to emerge. Interactions between fibronectin or galectin-8 and the inhibitory receptor LILRB4 have attracted particular interest because of their established roles in immune tolerance and cancer (Deng et al, 2018; Su et al, 2021; Wang et al, 2024). Although direct evidence linking these pathways to senescence remains limited, they highlight the growing appreciation that senescent cells may employ multiple strategies to modulate immune responses.

A broader realization emerging from these studies is that immune evasion may itself represent a hallmark of persistent senescent cells. Much like cancer cells, senescent cells appear capable of shaping their surrounding microenvironment in ways that reduce immune-mediated elimination. The balance between immune recognition and immune evasion, therefore, becomes a critical determinant of senescent-cell persistence and, ultimately, tissue health.

These findings shifted the field from a simple model of senescence surveillance toward a more dynamic model in which senescent cells and immune cells continuously interact through both activating and inhibitory signals. Understanding the mechanisms that govern this balance has become increasingly important, particularly as immune-based senotherapeutic strategies begin to emerge.

### Aging of the immune system and senescence accumulation

While immune-evasion mechanisms help explain the persistence of individual senescent cells, they do not fully account for the dramatic increase in senescent-cell burden observed during aging. A complementary explanation emerged from studies of immunosenescence, the progressive decline in immune function that accompanies aging (Nikolich-Žugich, 2018). This led to the hypothesis that senescent-cell accumulation reflects not only increased rates of senescence induction but also an age-dependent reduction in immune-mediated clearance.

Aging is associated with widespread alterations in both innate and adaptive immunity, including reduced NK-cell cytotoxicity, impaired macrophage function, diminished T-cell responses, and chronic low-grade inflammation, often referred to as inflammaging (Almeida-Oliveira et al, 2011; Britanova et al, 2014; Franceschi et al, 2000; Mahbub et al, 2012). These changes collectively compromise immune surveillance and may reduce the efficiency with which senescent cells are recognized and eliminated. Consistent with this idea, several studies have reported age-dependent defects in immune populations implicated in senescent-cell clearance, including NK cells, macrophages, and T lymphocytes (Ovadya and Krizhanovsky, 2014; Prata et al, 2018).

At the same time, senescent cells themselves can contribute to immune dysfunction through persistent SASP signaling. Chronic exposure to inflammatory cytokines, chemokines, and other SASP factors can alter immune-cell differentiation and function, creating a feed-forward cycle in which senescent cells promote immune dysfunction, which in turn further impairs their own clearance (Ovadya and Krizhanovsky, 2014; Prata et al, 2018). This reciprocal relationship has led to the concept that senescence and immunosenescence may reinforce one another during aging.

Although the relative contributions of increased senescence induction, immune evasion, and declining immune surveillance remain incompletely understood, an emerging view is that senescent-cell burden is determined by the balance between senescent-cell generation and removal. In young tissues, efficient immune surveillance may keep senescent cells transient and beneficial. During aging, however, persistent damage, immune dysfunction, and immune-evasion mechanisms may collectively shift this balance toward accumulation and chronic tissue dysfunction.

This has important therapeutic implications. Rather than focusing exclusively on eliminating senescent cells, interventions may also aim to restore immune-mediated clearance.

## From concept to therapy: senotherapeutics

The demonstration that senescent cells contribute causally to aging and age-related disease transformed senescence from a descriptive biological phenomenon into a potential therapeutic target. Early studies had largely focused on understanding how senescence is induced, maintained, and regulated. However, the discovery that elimination of senescent cells can improve tissue function and delay age-related decline raised a new possibility: could senescence itself be therapeutically manipulated?

Several observations supported this idea. Senescent cells accumulate with age, persist in multiple chronic diseases, and possess distinct molecular and metabolic features that differentiate them from their non-senescent counterparts. These properties suggested that senescent cells might be selectively targeted while sparing normal tissues. At the same time, growing recognition of the beneficial functions of senescence in tissue repair and development cautioned against indiscriminate elimination of all senescent cells.

**Figure 3:**
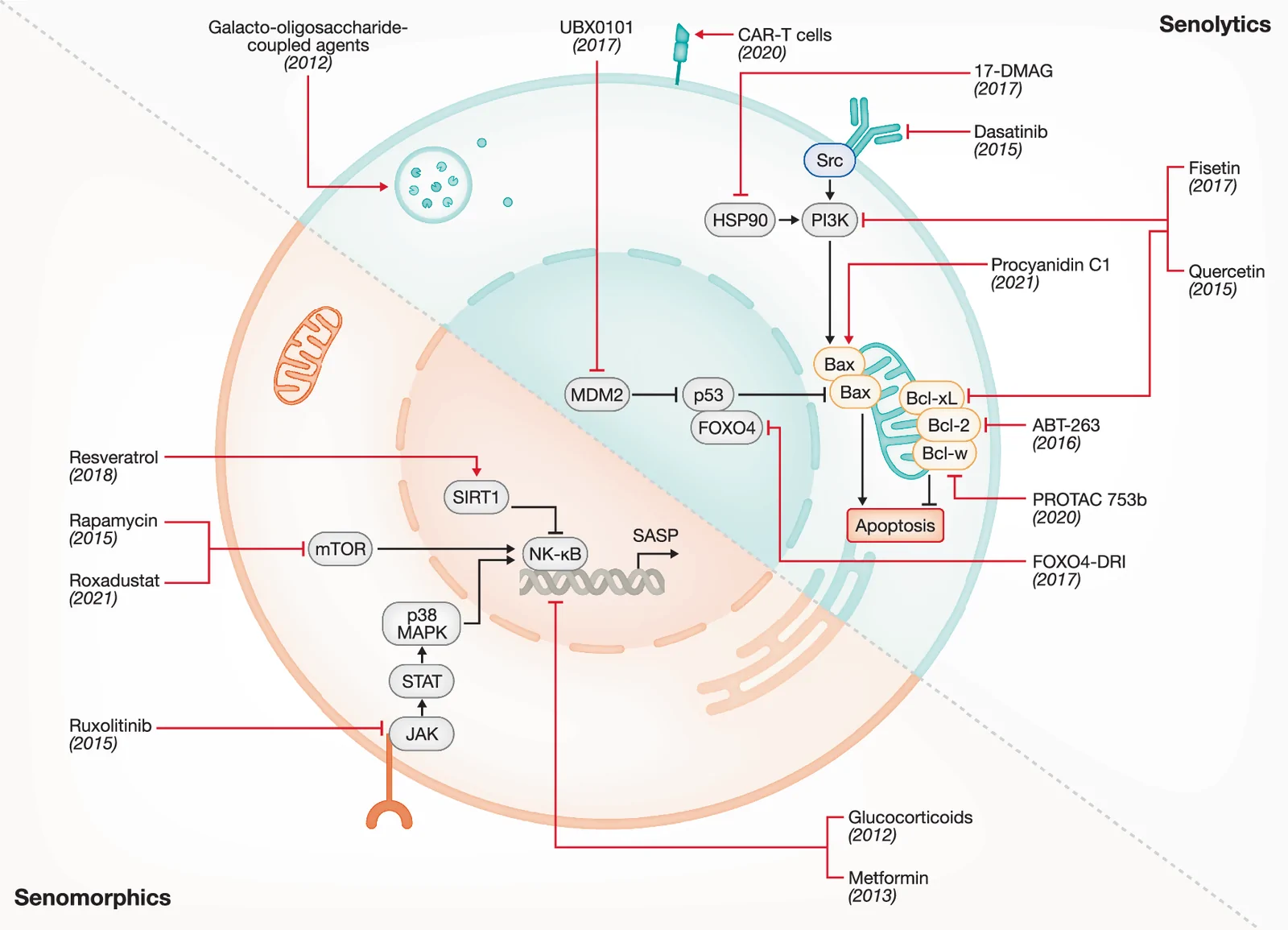
Therapeutic strategies targeting senescent cells. Senotherapeutic approaches can be broadly divided into senolytics and senomorphics. Senolytics selectively eliminate senescent cells by targeting survival pathways or exploiting senescence-associated vulnerabilities. Senomorphics suppress detrimental senescence-associated phenotypes, particularly inflammatory SASP programs, without necessarily eliminating senescent cells. Emerging precision approaches include immune-mediated clearance, targeted drug delivery, and interventions directed against specific senescent-cell subpopulations. Red arrows show how therapeutics target senescent cells. Black arrows show downstream pathways.

These considerations gave rise to the concept of senotherapeutics, a broad class of interventions designed to target senescent cells or their biological consequences (Fig. 3). Two major strategies subsequently emerged. The first, termed senolysis, aims to selectively eliminate senescent cells. The second, termed senomorphism, seeks to suppress the detrimental effects of senescent cells, particularly the SASP, without necessarily removing the cells themselves. Together, these approaches established a new translational dimension of senescence research and laid the foundation for the clinical development of therapies targeting fundamental mechanisms of aging.

### Senolytic strategies

The earliest senotherapeutic efforts focused on a simple but ambitious idea: if senescent cells actively contribute to aging and disease, their selective elimination might restore tissue function and improve health. This concept, termed senolysis, was inspired by genetic studies demonstrating that removal of senescent cells can delay age-related pathology and improve organismal function (Baker et al, 2016; Baker et al, 2011). The challenge was therefore to identify vulnerabilities that distinguish senescent cells from their normal counterparts and could be exploited therapeutically.

One of the first clues came from studies showing that senescent cells are remarkably resistant to apoptosis. As early as 1995, Wang et al reported elevated expression of anti-apoptotic BCL-2 family proteins in senescent fibroblasts (Wang, 1995). Subsequent work demonstrated that senescent cells depend on a network of pro-survival pathways, collectively termed senescent-cell anti-apoptotic pathways (SCAPs), to resist cell death despite extensive cellular damage and stress (Yosef et al, 2016; Zhu et al, 2015). These observations provided the conceptual foundation for the development of senolytic drugs.

A major breakthrough came in 2015 when Zhu et al used transcriptomic and functional screening approaches to identify the first senolytic drug combination capable of selectively eliminating senescent cells (Zhu et al, 2015). The combination of dasatinib and quercetin (D + Q) exploited distinct vulnerabilities across senescent cell types and demonstrated efficacy in multiple preclinical models. Subsequent studies showed that D + Q improves physical function, vascular health, and lifespan in aged mice, while also alleviating pathology in models of pulmonary fibrosis, metabolic dysfunction, and neurodegeneration (Ogrodnik et al, 2021; Palmer et al, 2019; Roos et al, 2016; Schafer et al, 2017; Xu et al, 2018; Zhang et al, 2019).

Shortly thereafter, navitoclax (ABT263), a BCL-2 family inhibitor targeting BCL-2, BCL-W, and BCL-XL, was identified as a second major senolytic strategy (Chang et al, 2016; Zhu et al, 2016). Although navitoclax effectively eliminated multiple senescent-cell populations and improved tissue function in several disease models, dose-limiting hematological toxicities highlighted one of the central challenges facing senolytic development: many senescent-cell survival pathways are shared with normal cells.

The field subsequently expanded beyond drug repurposing toward mechanism-based senolytic design. Approaches targeting FOXO4-p53 interactions (Baar et al, 2017), HSP90-dependent survival pathways (Fuhrmann-Stroissnigg et al, 2017), and BCL-family proteins through PROTAC technologies (He et al, 2020; Lv et al, 2021; Yang et al, 2025) demonstrated that senescent cells possess multiple targetable vulnerabilities. In parallel, increasing understanding of senescent-cell biology stimulated development of strategies exploiting unique senescence-associated features, including elevated lysosomal β-galactosidase activity for selective prodrug activation (Agostini et al, 2012; Cai et al, 2020; Muñoz-Espín et al, 2018).

Collectively, these studies established the senolytic concept: the idea that senescent cells can be selectively eliminated by exploiting vulnerabilities that arise as a consequence of the senescent state itself. This realization transformed senescence from a biomarker of aging into a pharmacologically actionable target and laid the foundation for the first generation of senotherapeutic interventions.

### Senomorphics

Although the emergence of senolytics generated considerable enthusiasm, growing appreciation of the physiological roles of senescence introduced an important caveat. Senescent cells contribute to tissue repair, regeneration, and developmental processes, raising concerns that indiscriminate elimination might also remove beneficial senescent populations. This realization stimulated the development of an alternative therapeutic strategy: rather than eliminating senescent cells, could their detrimental activities be selectively suppressed?

This concept gave rise to senomorphics, interventions that modulate the phenotype of senescent cells without necessarily inducing their death. Most senomorphic approaches emerged from efforts to suppress the SASP, which had become recognized as a major driver of chronic inflammation and age-related pathology. In contrast to senolytics, which target senescent-cell survival mechanisms, senomorphics aim to uncouple the detrimental consequences of senescence from the stable cell-cycle arrest that underlies many of its beneficial functions.

Early evidence supporting this concept came from observations that environmental conditions influence SASP composition. Culturing senescent cells under hypoxic conditions or exposing them to hypoxia mimetics resulted in a less inflammatory secretory phenotype (Coppé et al, 2010; van Vliet et al, 2021), demonstrating that the SASP is not a fixed property of senescence but a plastic and regulatable program.

Subsequent studies identified several signaling pathways that govern SASP production. Inhibition of p38 MAPK reduced inflammatory SASP components through suppression of MAPKAPK2 signaling (Alimbetov et al, 2016; Freund et al, 2011), while glucocorticoids such as corticosterone attenuated SASP through inhibition of the IL-1α/NF-κB axis (Laberge et al, 2012). Around the same time, metformin emerged as another candidate senomorphic through its ability to suppress NF-κB and JNK signaling pathways (Abdelgawad et al, 2023; Moiseeva et al, 2013).

A major advance came with the recognition that mTOR signaling serves as a central regulator of SASP production. Rapamycin was shown to suppress multiple pro-inflammatory cytokines, chemokines, and growth factors through effects on IL-1α translation, mTOR signaling, and MAPKAPK2 regulation (Herranz et al, 2015; Laberge et al, 2015). Similarly, inhibition of JAK signaling with ruxolitinib effectively reduced inflammatory SASP components and improved age-associated phenotypes in experimental models (Xu et al, 2015). Additional compounds, including resveratrol, have subsequently been reported to modulate SASP through regulation of NF-κB activity and related inflammatory pathways (Liu et al, 2018).

Together, these studies established that many of the detrimental effects of senescent cells can be pharmacologically separated from the senescent state itself. Rather than viewing senescence as an all-or-nothing target, senomorphic approaches introduced the possibility of selectively reshaping senescent-cell behavior.

However, senomorphic strategies also present unique challenges. Because these interventions do not necessarily reduce senescent-cell burden, they leave open the possibility that persistent senescent cells could regain detrimental activities once treatment is discontinued. Nevertheless, the senomorphic concept significantly broadened the therapeutic landscape by demonstrating that senescence can be manipulated through mechanisms other than cell elimination.

### Toward precision senotherapeutics

The first generation of senotherapeutics largely treated senescent cells as a single therapeutic target. However, advances in single-cell transcriptomics, spatial profiling, and genetic models have revealed extensive heterogeneity among senescent cells arising from different tissues, stimuli, and biological contexts (Hernandez-Segura et al, 2017; Wiley et al, 2017). This growing appreciation of senescence diversity is reshaping therapeutic strategies and challenging the notion that a single intervention will be effective across all senescence-associated conditions.

Consequently, the field is increasingly moving toward precision senotherapeutics, in which interventions are tailored to specific senescent-cell populations, tissues, or biological functions. Emerging approaches include targeted drug delivery systems, immune-based therapies such as CAR-T cells, antibody-mediated targeting, and interventions designed to selectively modulate distinct SASP programs (Amor et al, 2020; Campisi et al, 2019; Muñoz-Espín et al, 2018; Yoshida et al, 2020). Rather than simply eliminating all senescent cells, future therapies may seek to preserve beneficial senescent populations while selectively targeting those that drive pathology.

This shift mirrors a broader conceptual evolution within the field where senescence is not considered a uniform cellular state but a complex, heterogeneous and context-dependent biological process.

## Clinical translation: senotherapeutic trials

The translation of senotherapeutics from experimental concept to clinical reality has proven considerably more challenging than their rapid success in preclinical models initially suggested. While genetic and pharmacological studies established that senescent cells can drive age-related dysfunction in animals, demonstrating similar benefits in humans requires overcoming substantial biological, methodological, and regulatory hurdles. Consequently, the clinical history of senotherapeutics has thus far been characterized by a combination of early proof-of-concept successes, translational setbacks, and an increasing recognition of the need for robust biomarkers.

The first generation of clinical senotherapy largely relied on repurposing compounds originally developed for oncology and other indications. This strategy enabled rapid clinical testing by leveraging existing pharmacokinetic and safety data. A major milestone was reached in 2019 with the first published clinical senolytic study in patients with idiopathic pulmonary fibrosis (IPF), where intermittent treatment with dasatinib and quercetin (D + Q) was feasible and associated with improvements in selected measures of physical function (Justice et al, 2019). Shortly thereafter, a study in diabetic kidney disease provided the first tissue-level evidence that senolytic treatment could reduce senescence-associated markers, including p16, p21, and SA-β-gal activity in adipose tissue and skin biopsies (Hickson et al, 2019). Together, these studies established proof-of-concept that senescence-targeting interventions could be implemented in humans and that senescence-associated endpoints could be measured in clinical settings.

Subsequent studies expanded senotherapeutic testing into additional age-related conditions. A Phase 1 study in mild Alzheimer’s disease demonstrated that intermittent D + Q treatment was well tolerated and achieved measurable central nervous system exposure (Gonzales et al, 2023). Likewise, a randomized placebo-controlled pilot study in IPF further supported the feasibility and tolerability of D + Q treatment, although definitive conclusions regarding efficacy remained limited by sample size and study design (Nambiar et al, 2023). Additional compounds, including fisetin, have since entered early-stage clinical evaluation, although evidence for clinical benefit remains preliminary (Lee and Burns, 2024). In parallel, several interventions with potential senomorphic activity entered clinical testing through modulation of pathways such as mTOR, JAK/STAT, and inflammatory signaling. While these studies demonstrated that aging-associated pathways can be manipulated in humans, they generally did not directly assess senescent-cell burden, making it difficult to establish whether observed effects were mediated through senescence-related mechanisms (Kraig et al, 2018; Kulkarni et al, 2018; Vukmanovic-Stejic et al, 2018).

At the same time, clinical development revealed the difficulties of translating promising preclinical findings into meaningful patient benefit. One of the earliest examples was UBX0101, a senolytic developed for osteoarthritis that showed encouraging efficacy in preclinical models (Jeon et al, 2017). Despite these results, Phase 2 clinical studies failed to demonstrate significant clinical benefit, leading to discontinuation of the program. More recently, UBX1325 (foselutoclax), a BCL-xL-targeting senolytic developed for diabetic macular edema, showed favorable safety and early signs of biological activity (Crespo-Garcia et al, 2024; Klier et al, 2025), but subsequently failed to meet its primary endpoint in Phase 2b evaluation. These experiences highlighted a recurring challenge in the field: demonstrating senolytic activity in experimental systems does not necessarily translate into measurable clinical efficacy in humans.

These setbacks have increasingly shifted attention toward what is now widely recognized as the major bottleneck in clinical senotherapy: the lack of validated biomarkers capable of demonstrating target engagement in vivo. Commonly used markers, including p16, p21, SA-β-gal, and circulating SASP factors, provide useful information but lack sufficient specificity and validation to serve as definitive pharmacodynamic measures of senescent-cell burden. Consequently, a critical gap remains between demonstrating modulation of aging-associated pathways and confirming direct effects on senescent-cell populations. Addressing this challenge has become a major priority for the field, prompting increasing efforts toward biomarker standardization, cross-cohort validation, and the development of consensus frameworks for senescence measurement (Ogrodnik et al, 2024; Quarta et al, 2026; Suryadevara et al, 2024).

In this regard, the history of clinical senotherapy has increasingly become a history of biomarker development. However, improved measurement alone is unlikely to overcome the current translational challenges, and will need to progress alongside the development of more potent and selective senotherapeutics. These efforts will likely be iterative, with better biomarkers enabling more rigorous assessment of therapeutic activity and improved therapies, in turn, helping to validate biologically meaningful markers of senescence. The next generation of clinical trials will therefore need to combine meaningful clinical outcomes with rigorous mechanistic endpoints capable of linking therapeutic intervention directly to changes in senescence biology.

## Conclusions and future perspectives

**Figure 4:**
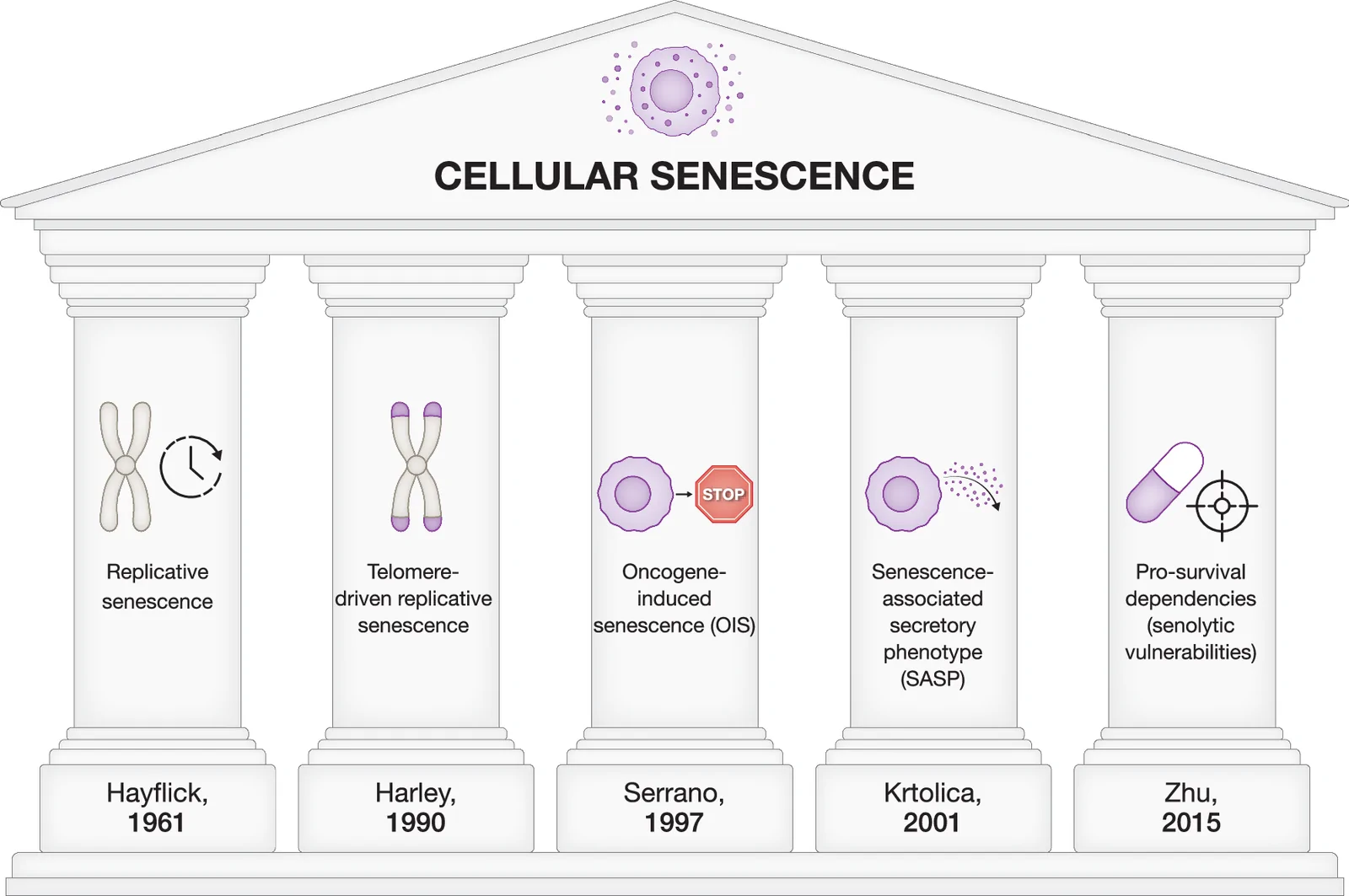
Conceptual evolution of the cellular senescence field. Summary of the major transitions that shaped modern understanding of senescence. Initially viewed as a consequence of replicative exhaustion, senescence subsequently emerged as a tumor-suppressive stress response, a tissue-remodeling program mediated by the SASP, a driver of aging and chronic disease, a process regulated by immune surveillance, and ultimately a therapeutic target. Recent advances in single-cell technologies have further revealed extensive heterogeneity among senescent cells, driving the development of precision senotherapeutic strategies.

The history of cellular senescence is, in many ways, a history of expanding biological complexity. What began as an observation of finite proliferative capacity in cultured cells has progressively evolved through a series of conceptual transitions that have fundamentally reshaped the field. Senescence was first understood as a consequence of replicative exhaustion, then as a potent tumor-suppressive mechanism, later as a regulator of tissue remodeling through the senescence-associated secretory phenotype, and ultimately as a causal contributor to aging and age-related disease. More recently, the emergence of senotherapeutics has transformed senescence from a biological phenomenon into a clinically actionable target (Fig. 4).

A major contemporary shift is the recognition that senescence is not a single cellular state. Advances in single-cell transcriptomics, spatial profiling, and lineage-specific genetic models have revealed extensive heterogeneity among senescent cells across different tissues, stimuli, and biological contexts. This emerging view is reshaping experimental design, biomarker discovery, and therapeutic development, while challenging many of the assumptions that historically treated senescence as a uniform biological process.

More than six decades after the original observations made by Hayflick, one of the most important lessons from the field is that senescence is neither inherently beneficial nor inherently detrimental. Rather, its biological consequences are determined by context. Senescent cells can promote tissue repair, regeneration, and tumor suppression, yet can also drive chronic inflammation, fibrosis, functional decline, and disease progression. Understanding this duality has become central to modern senescence biology.

This realization is driving a transition from broad senescent-cell elimination toward increasingly precise interventions tailored to specific senescent-cell populations and disease contexts. Future therapies will likely combine senolytic, senomorphic, immune-modulatory, and regenerative approaches to selectively suppress detrimental senescence programs while preserving or even harnessing beneficial ones. Achieving this goal will require robust biomarkers, standardized measurement frameworks, and improved patient stratification strategies capable of identifying when, where, and in whom senescence contributes to pathology.

Ultimately, the next phase of senescence research will depend less on determining whether senescence is “good” or “bad” and more on understanding which senescent cells, in which tissues, at which stage of life or disease, and through which mechanisms influence tissue function. The future of the field, therefore, lies not simply in eliminating senescent cells, but in deciphering and manipulating senescence with sufficient precision to restore tissue homeostasis, improve resilience, and promote healthy longevity.

## Supplementary information

Peer Review File
