# Supplementary material for: Cellular senescence: six decades of discovery and reinvention
Source: EMBO J. 2026 Oct 2;45(21):7307–25. doi: 10.1038/s44318-026-00922-w (PMC13728128; doi:10.1038/s44318-026-00922-w)
Supplement: Supplementary file 1 [file 44318_2026_922_MOESM1_ESM.pdf]

THE EMBO JOURNAL

Cellular Senescence: Six Decades of Discovery and Reinvention

Myrthe Klaver, Lotte Steeneken, Naomi Veeningen, Abdullah Altulea, and Marco Demaria

Corresponding author(s): Marco Demaria (m.demaria@umcg.nl)

Review Timeline:

Submission Date:	15th Jun 26
Editorial Decision:	30th Jul 26
Revision Received:	20th Aug 26
Accepted:	18th Sep 26

Editor: Daniel Klimmeck

Transaction Report:

(Note: With the exception of the correction of typographical or spelling errors that could be a source of ambiguity, letters and reports are not edited. Depending on transfer agreements, referee reports obtained elsewhere may or may not be included in this compilation. Referee reports are anonymous unless the Referee chooses to sign their reports.)

Dear Dr Demaria,

Thank you again for sending us your review article for consideration, as well as for your patience with our feedback at this time.

We have now received dedicated input on your manuscript from two senescence biology experts. As you will see, they much appreciate the review and find it timely and worth publishing pending minor amendments. Specifically, they provide constructive feedback on how to advance the literature context cited and complement the discussion, clarifying a number of aspects.

I hope you will find the comments helpful. I am sure that a final amended version incorporating the suggestions made by the referees will be highly noted and appreciated. I would thus like to invite you to submit such a revised version using the link enclosed below.

Further I also enclose below a number of formatting aspects to be considered at resubmission.

Please let me know in case I can be of any help with this.

Best regards,

Daniel Klimmeck

Daniel Klimmeck, PhD
Senior Editor
The EMBO Journal

Instructions for preparing your revised manuscript:

>> Add a DISCLOSURE AND COMPETING INTERESTS STATEMENT.
>> Please remove the figure drafts from the text and upload them as individual figure files.
>> Figure legends: please compile at the end of the manuscript text.
>> References: adjust to EMBO Journal style with 10 et al authors; remove dois.

Read our guidance for manuscript revisions and related editorial policies: https://link.springer.com/journal/44318/submission-guidelines#cms-Revised-submissions

----

Referee #1:

This is an excellent historical journey of the field of cellular senescence. I really enjoyed reading it! The paper focuses on the main conceptual leaps that have shaped this field. It flows nicely and logically, and it makes a great illustration of how scientific research changes our ideas and improves our understanding of biology, in health and disease. My most sincere congratulations to the authors.

I find the paper perfectly published as it is, text, figures, references.

I was taking notes while reading the paper and I realize that they are very minor. I just mention them here in case they are useful:

1. Subheading 3.1. When mentioning oncogenic signaling pathways that induce senescence, I think the first paper was PMID: 9765203 (from 1998).

2. Subheading 3.2. When mentioning ROS induced senescence, this paper (PMID: 12855956) was not the first but I think was very influential.

3. Subheading 5.3. I suggest to explicitly mention that examples of senescence in development have been reported across vertebrates, including fish and birds. This section or a different one could include the active role of senescence in blastema formation and organ regeneration, as it has been shown at least in salamanders (limb), fish (fins), and mice (digits). But the role of senescence in regeneration has been also shown in the spectacular example of cnidarians (PMID: 37392741), and even planarians (PMID: 26654402) activate senescence upon damage.

4. Subheading 7.3 "Aging of the Immune System Failure in Senescence Surveillance" reads a bit strange to me. I suggest: "Aging of the Immune System and Senescence Accumulation"

5. Subheading 9. I fully agree with the authors that more selective biomarkers of senescence are critical for the clinical advancement of senotherapies. In my opinion, but this is very personal, I think that the field also needs more potent therapies (higher efficiency and higher selectivity).

My congratulations again to the authors.

Referee #2:

This is a timely and comprehensive overview of the development of senescence research. The review effectively covers both the historical evolution of the field and future perspectives. I have only relatively minor suggestions.

Page 2. "...it shifted aging from being viewed solely as an organismal phenomenon to a process that could be studied at the cellular level." I am not sure this is entirely precise. I wonder whether the authors mean that these early studies represented an in vitro model of aging, or whether aging was simply interpreted that way at the time.

Page 4. PMID: 9765203 and 9765202: These two back-to-back papers were direct follow-up studies to the Serrano et al. paper and could also be cited.

Page 4. DNA damage signaling... (Bartkova et al., 2006; Di Micco et al., 2006): PMID: 17210786 could also be added.

Page 4. OIS: It would be worth mentioning the recent study (PMID: 39112713), which addresses the longstanding apparent conflict between oncogene-induced senescence (OIS) and tumour initiation by introducing the concept of a senescence spectrum, highlighting the heterogeneity of OIS.

Page 7. "Mechanistically, NF-κB emerged as one of the first master regulators of inflammatory SASP programs (Chien et al., 2011)." The authors could also consider the earlier studies (PMID: 18555778 and 18555777) and mention C/EBPβ as a key transcription factor that cooperates with NF-κB in regulating the SASP.

Page 7. cGAS... The recent study (PMID: 39191740) identified an additional cytoplasmic nucleic acid-sensing mechanism (mt-dsRNA detection by RIG-I/MDA5-MAVS) that regulates the SASP alongside the cGAS-STING pathway.

Page 9. The study by Chuprin et al. (PMID: 24186980) could be included as another early example of senescence in development.

Page 10. The INK-ATTAC mouse model demonstrated beneficial effects of eliminating p16-expressing cells on ageing. It would also be worth mentioning another p16-lineage elimination model, which reported detrimental effects of p16-lineage cell ablation (PMID: 32485135).

We would like to thank the reviewers for their kind words and, above all, for the time and care they took in reading our manuscript and providing insightful comments. All comments are addressed below.

Referee #1:

This is an excellent historical journey of the field of cellular senescence. I really enjoyed reading it! The paper focuses on the main conceptual leaps that have shaped this field. It flows nicely and logically, and it makes a great illustration of how scientific research changes our ideas and improves our understanding of biology, in health and disease. My most sincere congratulations to the authors.

I find the paper perfectly published as it is, text, figures, references.

I was taking notes while reading the paper and I realize that they are very minor. I just mention them here in case they are useful:

1. Subheading 3.1. When mentioning oncogenic signaling pathways that induce senescence, I think the first paper was PMID: 9765203 (from 1998).
We appreciate the reviewer for bringing this paper to our attention. Upon reviewing it, we have incorporated the corresponding reference into the text.

2. Subheading 3.2. When mentioning ROS induced senescence, this paper (PMID: 12855956) was not the first but I think was very influential.
We thank the reviewer for drawing our attention to this paper, which we have now cited in the text accordingly.

3. Subheading 5.3. I suggest to explicitly mention that examples of senescence in development have been reported across vertebrates, including fish and birds. This section or a different one could include the active role of senescence in blastema formation and organ regeneration, as it has been shown at least in salamanders (limb), fish (fins), and mice (digits). But the role of senescence in regeneration has been also shown in the spectacular example of cnidarians (PMID: 37392741), and even planarians (PMID: 26654402) activate senescence upon damage.
We want to thank the reviewer for this insightful comment. We expanded the section to include senescence in development across different species. The extended text in subsection 5.3 reads: Subsequent studies identified developmental senescence in additional vertebrate species, including birds, amphibians and fish, suggesting that senescence during embryogenesis represents an evolutionary conserved program rather than a species-specific phenomenon (Da Silva-Álvarez *et al*, 2020b; Davaapil *et al*, 2017; Nacher *et al*, 2006; Villiard *et al*, 2017).
For the role of senescence in regeneration, we reviewed the mentioned papers and added additional references to subsection 5.2. However, it needs to be mentioned that in the paper about planarians (PMID: 26654402), the evidence on the role of senescence is limited. Therefore, we mention the paper with a critical note. The new text of this subsection now reads: “Subsequent studies extended these findings across vertebrate and

invertebrate organisms, demonstrating functional roles for senescence in tissue regeneration and responses to injury, including during limb and fin regeneration (Da Silva-Álvarez *et al*, 2020a; Salinas-Saavedra *et al*, 2023; Yun *et al*, 2015). Nevertheless, in certain models, such as planarians, the evidence remains limited, and additional markers as well as further research are required before the role of senescence can be conclusively established."

4. Subheading 7.3 "Aging of the Immune System Failure in Senescence Surveillance" reads a bit strange to me. I suggest: "Aging of the Immune System and Senescence Accumulation"
The reviewer is correct that the suggested heading reads more clearly. We changed the original heading to the suggested heading of the reviewer.

5. Subheading 9. I fully agree with the authors that more selective biomarkers of senescence are critical for the clinical advancement of senotherapies. In my opinion, but this is very personal, I think that the field also needs more potent therapies (higher efficiency and higher selectivity).
We thank the reviewer for this important comment, and we fully agree with it as well. The development of high specificity senotherapeutic agents represents an equally important challenge for the field as biomarker development. In practice, these two challenges are closely linked and will likely need to be addressed in parallel. We believe it will take several rounds of refinement on both sides before robust and functionally relevant senescent-cell clearance can be demonstrated in humans. We adjusted the last paragraph of subheading 9 to better reflect these points. This part now reads: "In this regard, the history of clinical senotherapy has increasingly become a history of biomarker development. Future progress will likely depend as much on improving the measurement of senescence as on the development of new therapeutic agents. However, improved measurement alone is unlikely to overcome the current translational challenges, and will need to progress alongside the development of more potent and selective senotherapeutics. These efforts will likely be iterative, with better biomarkers enabling more rigorous assessment of therapeutic activity and improved therapies, in turn, helping to validate biologically meaningful markers of senescence. The next generation of clinical trials will therefore need to combine meaningful clinical outcomes with rigorous mechanistic endpoints capable of linking therapeutic intervention directly to changes in senescence biology."

My congratulations again to the authors.

Referee #2:

This is a timely and comprehensive overview of the development of senescence research. The review effectively covers both the historical evolution of the field and future perspectives. I have only relatively minor suggestions.

Page 2. "...it shifted aging from being viewed solely as an organismal phenomenon to a process that could be studied at the cellular level." I am not sure this is entirely precise. I wonder whether the authors mean that these early studies represented an in vitro model of aging, or whether aging was simply interpreted that way at the time.
We thank the reviewer for this critical assessment of the text. We modified the text, which now reads: "More importantly, the discovery established replicative senescence as an experimental paradigm through which cellular mechanisms contributing to aging could be investigated."

Page 4. PMID: 9765203 and 9765202: These two back-to-back papers were direct follow-up studies to the Serrano et al. paper and could also be cited.
We thank the reviewer for drawing our attention to these papers. We reviewed the papers and incorporated the references to the text accordingly.

Page 4. DNA damage signaling... (Bartkova et al., 2006; Di Micco et al., 2006): PMID: 17210786 could also be added.
We appreciate the reviewer bringing this paper to our attention and have cited it accordingly in the revised text.

Page 4. OIS: It would be worth mentioning the recent study (PMID: 39112713), which addresses the longstanding apparent conflict between oncogene-induced senescence (OIS) and tumour initiation by introducing the concept of a senescence spectrum, highlighting the heterogeneity of OIS.
We want to thank the reviewer for this important observation. We have reviewed the mentioned paper and expanded our section on OIS. The new text now reads: "Collectively, these discoveries fundamentally redefined senescence biology by positioning senescence as an intrinsic tumor-suppressive mechanism and one of the first examples of a cellular response capable of actively preventing malignant transformation. More recent recognition of OIS heterogeneity has further refined this paradigm, emphasizing that the biological consequences of OIS are highly context dependent."

Page 7. "Mechanistically, NF-κB emerged as one of the first master regulators of inflammatory SASP programs (Chien et al., 2011)." The authors could also consider the earlier studies (PMID: 18555778 and 18555777) and mention C/EBPβ as a key transcription factor that cooperates with NF-κB in regulating the SASP.
We thank the reviewer for this constructive suggestion. We have now mentioned C/EBPβ in the text together with the corresponding references. The new text reads: "Mechanistically, NF-κB and C/EBPβ emerged as one of the first master major transcriptional regulators of inflammatory SASP programs (Acosta *et al.*, 2008; Chien *et al.*, 2011; Kuilman *et al.*, 2008)."

Page 7. cGAS... The recent study (PMID: 39191740) identified an additional cytoplasmic

nucleic acid-sensing mechanism (mt-dsRNA detection by RIG-I/MDA5-MAVS) that regulates the SASP alongside the cGAS-STING pathway.

We agree with the reviewer that this paper is important to reference. Upon reviewing the paper, we added the RNA sensing mechanism to the main text. The reviewed part of the text now reads: “The cGAS-STING pathway emerged as an additional mechanism linking persistent DNA damage to inflammatory and interferon responses through the detection of cytoplasmic DNA sensing, while more recent work identified mitochondrial double stranded RNA sensing via the RIG-I/MDA5-MAVS pathways as a complementary regulator of SASP expression chromatin fragments and micronuclei (Dou *et al*, 2017; Glück *et al.*, 2017; López-Polo *et al*, 2024; Yang *et al*, 2017).”

Page 9. The study by Chuprin et al. (PMID: 24186980) could be included as another early example of senescence in development.

We thank the reviewer for suggesting this paper. The text was updated to the following: “These findings, together with complementary evidence from Chuprin et al. demonstrating the role for senescence in embryonic tissue remodeling (Chuprin *et al*, 2013), challenged the prevailing view that senescence was solely a response to cellular stress, DNA damage, oncogenic signaling, or aging-associated dysfunction.”

Page 10. The INK-ATTAC mouse model demonstrated beneficial effects of eliminating p16-expressing cells on ageing. It would also be worth mentioning another p16-lineage elimination model, which reported detrimental effects of p16-lineage cell ablation (PMID: 32485135).

We agree with the reviewer that it is worth mentioning this other p16-lineage elimination model. We have read the suggested paper and included their findings. The new text now reads as follows: “The strongest evidence for a causal role of senescence came from genetic ablation studies. Using INK-ATTAC mice, Baker and colleagues demonstrated that removal of p16-positive cells delays age-associated pathology in progeroid animals (Baker *et al.*, 2011). Subsequent studies extended these findings to naturally aged mice and showed improvements in healthspan and lifespan following senescent-cell clearance (Baker *et al.*, 2016). However, these effects were not universally reproduced across senescent-cell ablation models. An independent p16-lineage tracing and ablation study reported that elimination of p16-expressing cell lineages can have detrimental effects on tissue function and organismal health, highlighting the context dependence of senescent cell contributions to aging (Grosse *et al*, 2020). These experiments fundamentally changed the field by demonstrating that senescent cells are not merely passive indicators of aging but active contributors to age-related decline, while also revealing that their effects can depend on the cellular population targeted and the experimental context.”

Dear Dr Demaria,

Thank you for submitting the revised version of your review manuscript for consideration by the EMBO Journal.

I have now carefully checked your amendments towards the experts' comments and found them to be well addressed and plausibly integrated into the revised text. I am thus very pleased to inform you that your review article has now been accepted for publication in the EMBO Journal.

Your manuscript will be processed for publication by EMBO Press. It will be copy edited and you will receive page proofs prior to publication. Also, you will soon be contacted by Springer Nature to sign your publishing license.

Should you experience any difficulty, please email publishing@embo.org.

If you have any questions, please do not hesitate to contact me or the Editorial Office.

I look forward to progressing swiftly towards online publication of this review article!

with
Best wishes,

Daniel Klimmeck

Daniel Klimmeck, PhD
Senior Editor
The EMBO Journal
EMBO
Postfach 1022-40
Meyerhofstrasse 1
D-69117 Heidelberg
contact@embojournal.org
Submit at: http://emboj.msubmit.net

*****
Please note that it is The EMBO Journal policy for the transcript of the editorial process (containing referee reports and your response letters) to be published as an online supplement to each paper. If you should prefer removal of any referee-only figures included in the point-by-point response(s), e.g. because they may still be used for future publication or because they have been reproduced from published work by others, please do let us know immediately via response email.
More information is available here: https://link.springer.com/partners/embo-press/editorial-policies#Peer%20review

** Click here to be directed to your login page: https://emboj.msubmit.net
